# Pharmacologic Management of Monogenic and Very Early Onset Inflammatory Bowel Diseases

**DOI:** 10.3390/pharmaceutics15030969

**Published:** 2023-03-17

**Authors:** Anne E. Levine, Dominique Mark, Laila Smith, Hengqi B. Zheng, David L. Suskind

**Affiliations:** 1 Division of Gastroenterology, Seattle Children’s Hospital, Seattle, WA 98105, USA; 2 Department of Pediatrics, University of Washington School of Medicine, Seattle, WA 98195, USA; 3 Department of Pharmacy, Seattle Children’s Hospital, Seattle, WA 98105, USA

**Keywords:** very early onset inflammatory bowel disease (VEO-IBD), pharmacotherapy, monogenic IBD, management

## Abstract

Inflammatory bowel disease (IBD) is treated with a variety of immunomodulating and immunosuppressive therapies; however, for the majority of cases, these therapies are not targeted for specific disease phenotypes. Monogenic IBD with causative genetic defect is the exception and represents a disease cohort where precision therapeutics can be applied. With the advent of rapid genetic sequencing platforms, these monogenic immunodeficiencies that cause inflammatory bowel disease are increasingly being identified. This subpopulation of IBD called very early onset inflammatory bowel disease (VEO-IBD) is defined by an age of onset of less than six years of age. Twenty percent of VEO-IBDs have an identifiable monogenic defect. The culprit genes are often involved in pro-inflammatory immune pathways, which represent potential avenues for targeted pharmacologic treatments. This review will provide an overview of the current state of disease-specific targeted therapies, as well as empiric treatment for undifferentiated causes of VEO-IBD.

## 1. Introduction

Inflammatory bowel disease (IBD), including ulcerative colitis (UC), Crohn’s disease (CD), and IBD-unspecified (IBD-U), is a chronic, relapsing intestinal disease characterized by inflammation. The pathogenesis involves a complex interplay of the intestinal immune system and gut microbiome, as well as environmental factors. IBD is a heterogeneous disease with a wide variety of presentations. The majority of patients have what is considered polygenic IBD, in that their disease is a result of the interplay of many genes with environmental factors. Patients who present before the age of six years are designated as having very early onset IBD (VEO-IBD), which can be further subclassified into infantile IBD, for children diagnosed before two years of age, and neonatal IBD, for those diagnosed before 28 days of life [1]. In contrast to older children and adolescents with polygenic IBD, patients with VEO-IBD often have recalcitrant disease course and are more likely to have a causative monogenic defect underlying their disease [1,2]. Recent studies suggest that a monogenic immunodeficiency may be identified in 20–30% of patients with VEO-IBD (Table 1) [3,4]. These patients are usually identified during early life. In an increasing number of cases, these monogenic immunodeficiencies can be treated with targeted pharmacologic agents and other therapies, such as stem cell transplantation. The armamentarium of biologic and small molecule therapies continues to grow, which allows for more narrowly targeted treatments even in undifferentiated VEO-IBD. As the incidence of pediatric IBD, and with it VEO-IBD, continues to rise, identifying patients with monogenic immunodeficiencies becomes ever more urgent to target treatment and minimize medication toxicities [5,6]. This review will provide an overview of the current state of pharmacologic therapies for monogenic IBD, as well as empiric therapies for undifferentiated VEO-IBD.

## 2. Methods

A comprehensive search of MEDLINE limited to English language articles was performed using PubMed (http://pubmed.ncbi.nlm.gov) accessed on 4 January 2023. The following search terms were used: “very early onset inflammatory bowel disease”, “monogenic inflammatory bowel disease”, as well as gene names, syndrome names, and medication names (both brand and generic). Articles were used if they were peer-reviewed and included clinical information about monogenic or very early onset IBD cases, or if they discussed the use of a medication to target a specific immunologic pathway.

## 3. Monogenic Inflammatory Bowel Diseases

Currently, over 100 disease-associated genes have been identified [1,7,8]. Causative immunodeficiencies can be classified as epithelial cell defects, phagocytic defects, defects of adaptive immunity, T regulatory defects, IL-10 pathway disorders, and hyperinflammatory or autoinflammatory conditions [3,9,10,11,12] (Table 1). Monogenic defects of the epithelium often involve the gut and the skin due to shared embryologic precursors. Defects of phagocytosis lead to defective degranulation, as in chronic granulomatous disease (CGD) [13,14], or impaired migration of neutrophils into the tissue in leukocyte adhesion disorder (LAD) [15,16,17]. T or B cell defects that result in severe combined immunodeficiency (SCID) or common variable immunodeficiency (CVID) often present with an IBD-like phenotype [18,19,20,21,22]. Wiskott-Aldrich syndrome [23], or X-linked agammaglobulinemia [24], can also have an IBD-like component. IBD is a common presentation of T regulatory defects, such as immunodysregulation, polyendocrinopathy, enteropathy X-linked syndrome (IPEX), or IPEX-like syndromes [25,26,27,28,29,30]. Hyperinflammatory conditions, such as X-linked inhibitor of apoptosis (XIAP), often present with a severe perianal Crohn’s disease phenotype [31,32]. Finally, loss of function mutations in the IL-10 ligand and receptor can cause a broad spectrum of IBD-like disease [33,34,35].

## 4. Diagnosing Monogenic Inflammatory Bowel Disease

As more genes are identified and new therapeutics continued to be developed, a high level of suspicion for monogenic disorders should be maintained in patients with both VEO-IBD and severe refractory disease at older ages. Appropriate testing should be performed with the assistance of a multidisciplinary team, including pediatric gastroenterologists, geneticists, and immunologists [12]. Other team members may include dieticians, psychologists, and social workers to support the complex needs of these patients and their families. A thorough evaluation should be performed in these patients, including a comprehensive history with focus on family and genetic history, physical exam, blood work and stool studies, endoscopy, histology, and genetic work up. First-line laboratory evaluation should include a complete blood count (CBC) with differential, comprehensive metabolic panel, inflammatory markers with C-reactive protein (CRP) and erythrocyte sedimentation rate (ESR), and stool evaluation to rule out infection. Several diagnostic algorithms now exist to guide immunologic work up, which may include immunoglobulin levels, vaccine response titers, and flow cytometry of B, T, and NK cell populations. Specific disorders may be diagnosed via neutrophil oxidative burst (CGD), gene panels, or targeted flow cytometry (IPEX due to *FOXP3* mutation, XIAP), or cytokine panels [3,12]. Upper endoscopy and colonoscopy with histologic evaluation is a standard diagnostic evaluation in cases of suspected IBD. Findings in the VEO and monogenic IBD population are often non-specific, although the presence of certain histologic findings, including apoptosis, severe chronic architectural changes, villous blunting, and abundance of eosinophils, should increase the index of suspicion for a monogenic disorder [9,36].

Currently, approximately 20% of children with VEO-IBD have an underlying genetic disorder identified, although a recent study of 207 patients with VEO-IBD aimed at defining the role of next generation sequencing showed that a molecular diagnosis was achieved 32% of the time [4]. If patients primarily had small bowel inflammation, the yield increased to 61%. For those patients with colitis and perianal lesions, the yield was 39%, and it was 18% for colitis only [4]. Monogenic IBD has also been found with increased prevalence in older children and adolescents with severe, refractory IBD, with some cohorts finding monogenic disorders in up to 30% of older patients with refractory disease [37]. In the future, there is potential for the use of polygenic risk scores (PRS) to predict risks of disease progression and severity in these patients. Adult studies have shown that PRS can help identify patients at risk of developing stenotic disease requiring ileocecal resection or primary sclerosing cholangitis (Voskuil 2021 930) [38]. Another PRS replicated the association of two VEO-IBD genes, *ADAM17* and *LRBA*, and showed that heterozygous carriage of a specific mutant *LRBA* allele is also associated with significantly decreased LRBA and CTLA-4 expression with T-cell activation [39]. Currently, the applicability of PRS is limited by low discriminative accuracy due to small population sizes, and they cannot account for environmental factors or microbiome interplay.

As monogenic disorders are inborn errors of immunity leading to dysfunction in specific immune pathways with known alterations in cytokine activity, treatment teams are increasingly choosing targeted pharmacologic agents, rather than empiric IBD therapies in these patients. In the absence of a treatment-guiding diagnosis, children with undifferentiated VEO-IBD are treated empirically with corticosteroids, 5-aminosalicylates, immunomodulators, biologics, small molecules, and antibiotics [10,40]. Severe, refractory disease is more common in VEO-IBD [3], and management often includes non-pharmacologic therapies, including nutrition and surgery. In some cases, children with underlying monogenic defects also benefit from hematopoietic stem cell transplant (HSCT) to cure their immunodeficiency [41].

## 5. Targeted Therapies for Monogenic IBD

### 5.1. Anti-TNF Antibodies

Monoclonal antibodies against tumor necrosis factor alpha (TNFα) include infliximab, adalimumab, and golimumab. Infliximab has been trialled to target TNF-driven IBD-like inflammation seen in X-linked ectodermal dysplasia and immunodeficiency, an epithelial cell defect secondary to mutations in *IKBKG/NEMO*, in which defective NF-κB activation impairs immune response to circulating TNF [42], as well as Hermansky-Pudlak syndrome, a hyperinflammatory disorder involving defective cellular trafficking of lysosome-related organelles [43]. Studies of infliximab in these disorders have not been performed, but case reports show promise. A patient with X-linked ectodermal dysplasia showed good response in one report [44], and another with Hermansky-Pudlak syndrome and Crohn’s-like colitis remained in long-term remission at 22 months of treatment [45]. In another case report, an adult patient with incomplete IPEX syndrome who was treated with infliximab had improvement in clinical symptoms and inflammatory markers, as well as increase in circulating FOXP3+ CD4+ T regulatory cells, suggesting a role for anti-TNF therapy in potentiating T regulatory cell expansion and activity in this disorder [46]. However, infliximab should not be used in patients with chronic granulomatous disease, as it has been linked with severe infections and death [47].

#### 5.1.1. Pharmacologic Considerations and Therapeutic Drug Monitoring for Anti-TNFα Therapy

Anti-TNFα agents are widely used as first-line biologic therapy in undifferentiated pediatric IBD and VEO-IBD. Infliximab has a remission rate of 60% by week 10 of treatment in children, with up to 65% maintaining remission at one year [48]. The clinical response to infliximab is variable in both adults and children, and therefore the role of therapeutic drug monitoring (TDM) has become the standard of care in IBD across both populations [49]. It is expected that up to one third of patients will require dose escalation within the first year of treatment due to secondary loss of response [50]. Infliximab requires therapeutic drug level monitoring to ensure that patients maintain therapeutic levels and to monitor for development of anti-infliximab antibodies that can lead to loss of response [51]. Younger children also have increased clearance of infliximab and may require higher doses or shorter intervals to maintain therapeutic levels [51,52,53,54]. Concurrent use of an immunomodulator, such as azathioprine or methotrexate, has been shown to reduce immunogenicity, leading to higher drug levels, reduced antibody formation, and endoscopic improvement [55,56]. However, the use of azathioprine in particular as combination therapy has been linked to the development of uniformly fatal hepatosplenic T-cell lymphoma primarily in teenage males, and thus duration of therapy is often limited to two years or less [10].

There is no consensus on the role of proactive TDM as compared to reactive monitoring; however, the literature supports the role of TDM in maintaining optimal treatment effect [57]. Recent recommendations from the ECCO-ESPGHAN group suggest early proactive TDM to optimize drug dosing [49]. Trough levels are typically checked during the maintenance phase of therapy, as compared to the induction phase. A trough level of >5 mcg/mL is preferred. However, in perianal fistulizing disease, a target trough level of >12.7 mcg/mL has been correlated with better clinical response (Table 2).

Recommendations regarding the use of adalimumab in undifferentiated pediatric IBD suggest that it can also be considered as a first line-agent. It remains an effective option in both biologic naïve patients, as well as those who have had previous biologic exposure. Trough levels are checked typically during the maintenance of treatment, with a goal level of at least 7.5 mcg/mL recommended for endoscopic healing by week eight of therapy [49] (Table 2).

#### 5.1.2. Time to Therapeutic Effect

Anti-TNFs can require up to 12 weeks to see full clinical effect, and response can be variable and patient specific; however, data in the adult population suggest that up to 81% of patients with Crohn’s disease will have evidence of clinical response after four weeks of infliximab therapy [77,78,79]. Adult response to adalimumab varies from 12 to 15 weeks in Crohn’s disease, and as early as four weeks in the UC population [79,80,81]. In pediatric UC patients, one study showed that up to 75% of patients with effective response to infliximab treatment by week eight of therapy [82]. However, loss of response is quite common, with up to one third of patients on infliximab experiencing secondary loss of response [50]. For pediatric patients treated with adalimumab, clinical remission rates range from two weeks to two years [83].

### 5.2. Vedolizumab

Vedolizumab is a gut-specific anti-α4β7 integrin monoclonal antibody that prevents migration of T cells into colonic tissue, thereby reducing intestinal inflammation. It has been used with varying degrees of success for certain monogenic IBD variants, including chronic granulomatous disease and CTLA4 deficiency. There is a report of sustained remission of CGD colitis and perianal disease in an adult [84]; however, the pediatric data are less promising. In a study of 11 children, more than half had symptomatic improvement and many were able to wean steroids, but none achieved mucosal healing after six months of treatment [85]. Vedolizumab has also been trialled for CTLA4 deficiency, and in a case report of a single adult patient, there was sustained remission; however, pediatric studies of this disorder are lacking, and it is not generally recommended as a first-line agent in this disorder [86]. It is, however, safe and effective in undifferentiated VEO-IBD with a 56% clinical response rate by the fourth dose [87].

#### 5.2.1. Pharmacologic Considerations and Therapeutic Drug Monitoring for Vedolizumab

Vedolizumab has a low incidence of adverse drug reactions reported in the literature, and anti-drug antibody production is relatively rare [49,88] (Table 3). From a TDM perspective, there is not clear guidance on optimal trough levels or when to check them. In one pediatric study, average trough levels of 32.1 mcg/mL were observed at week two of therapy and 29.9 mcg/mL at week six in a cohort of 22 pediatric patients. When this population was further delineated by type of IBD, trough levels in patients with UC/IBD-U were higher than in those with CD [66] (Table 2).

#### 5.2.2. Time to Therapeutic Effect

Pediatric studies indicate that vedolizumab may be more effective in UC compared to CD, and patients without prior biologic exposure and less severe disease may have higher rates of both mucosal healing and clinical remission [104]. One study showed that response to vedolizumab may take up to 16 weeks, with a range of therapeutic onset between eight and twelve weeks. In a pediatric retrospective study, 76% of those with UC compared to 42% of those with CD achieved clinical remission after 14 weeks of treatment [105] (Table 4).

### 5.3. Ustekinumab

Ustekinumab is a humanized monoclonal anti-IL-12/23 antibody used in pediatric patients who are non-responsive to anti-TNF medications. While it has been shown to be safe and efficacious in both biologic-exposed and naïve patients, its use in the VEO-IBD and monogenic IBD population is limited, and there are no large-scale trials [112,113]. A single case report in a patient with VEO-IBD due to Loeys-Dietz syndrome reported clinical remission with a trough level of 6 mcg/mL after 18 months of treatment [68]. The authors posited that the patient’s colitis could have been the result of overactivation of TH17 cells secondary to the underlying mutation in TGF-β, resulting in high levels of IL-23.

#### Pharmacologic Considerations and Therapeutic Drug Monitoring for Ustekinumab

Studies have shown that ustekinumab is generally well tolerated, but it still holds an increased risk of infection and hypersensitivity reactions during administration (Table 3). When evaluating the use of ustekinumab in pediatric IBD, there has been some evidence to show better response and higher rates of clinical remission in patients who are biologic naïve [113]. In a recent retrospective study conducted in the pediatric population, 64% of patients achieved biomarker remission at the 52-week mark, with 76% of patients still remaining on ustekinumab at the end of the study period. From this cohort of patients, 58% achieved steroid free clinical remission [113]. In adult trials, clinical response was seen after eight weeks of treatment, with some data to suggest a duration of up to six months needed for endoscopic remission [67] (Table 4). In the future, these patients may also be candidates for treatment with selective anti-IL-23 agents, including risankizumab, which is approved for adults with Crohn’s disease, and mirikizumab, which is approved for adults with ulcerative colitis.

### 5.4. Janus Kinase Inhibitors

Ruxolitinib and tofacitinib inhibit JAK1/2 and JAK1/3, respectively, and via this pathway downregulate several inflammatory cytokines, including IL-6, IL-11, IL-4, IL-7, and IL-9 [51]. There have been several case studies showing good response to these agents in patients with mutations in both *JAK* and *STAT* genes, which lead to IPEX-like syndromes [114]. A patient with a *STAT3* gain-of-function mutation who was refractory to multiple previous therapies had sustained remission at one year on ruxolitinib [115]. IL2RA mutations are another target for Janus kinase inhibitors, as a key component of the IL-2R signaling pathway is encoded by the STAT5B gene [116,117]. In vitro exposure to tofacitinib of resected colonic tissue from a patient with refractory colitis due to *IL2RA* IPEX-like syndrome led to reduction in IL-2 and IFNγ secretion [118].

#### Pharmacologic Considerations and Therapeutic Drug Monitoring for JAK Inhibitors

Janus kinase inhibitors are small molecules and thus do not have the same risk of loss of response due to immunogenicity as the anti-TNFs. They are available in oral form and do not require drug level monitoring. Tofacitinib is approved for use in adults with ulcerative colitis; off-label use in pediatric UC also shows promise [119]. In a single-center, retrospective study, Tofacitinib induced clinical remission in nearly 50% of tested patients after the 12-week induction period (N = 9/21). After 52 weeks, approximately 40% of subjects achieved steroid free remission (N = 7/17) [119]. Of note, 67% of patients included in this study had ulcerative colitis, 14% had Crohn’s disease, and 19% had indeterminant IBD. More selective agents, such as upadicitinib, a JAK1 inhibitor, are coming to market, but they are not yet approved for use in children. Risks associated with the use of JAK inhibitors include anemia due to inhibition of erythropoietin signaling and venous thromboembolism [120]. From a monitoring standpoint, patients receiving JAK inhibitors should have lipids, CBC, renal function, and liver function assessed prior to initiating therapy and periodically while on maintenance (Table 3) [102,103].

### 5.5. IL-1 Antagonists

There are two recombinant IL-1 inhibitors available, canakinumab and anakinra, both of which act by competitive inhibition of the IL-1 receptor. IL-1 release is triggered by activation of the inflammasome, and it leads to a proinflammatory cascade [121,122]. Several monogenic disorders involve activation of the inflammasome and subsequent IL-1 secretion, including CGD and mevalonate kinase deficiency (MKD). Patients with chronic granulomatous disease have defective autophagy, which has been linked to dysregulated activation of the inflammasome [123]. A small number of case studies using anakinra in patients with CGD enterocolitis have shown a modest response, though with low rates of long-term remission [123,124]. Successful use of anakinra ranging from partial symptomatic improvement to remission has been reported in several case series of infants with MKD, an autoinflammatory disorder that often presents in infancy with IBD-like enterocolitis, and it also involves inflammasome-driven IL-1β secretion [125,126]. In a recent study, a cohort of nineteen children with VEO-IBD (approximately 50% with infantile onset, 42% with Crohn’s disease, and 58% with unclassified IBD) received canakinumab with some success. These patients had an autoinflammatory phenotype without monogenic disease and were treated with canakinumab therapy for at least six months. At baseline, close to 40% of patients were biologic naïve; in 74% of the studied population, canakinumab was used in dual therapy with either anti-TNF alpha blockers, vedolizumab, ruxolitinib, ustekinumab, or rapamycin. Clinical remission was achieved in 32% of patients after six months of treatment, and clinical response was achieved in 89% of patients (17/19) [127,128]. Additionally, in a 2020 study, which included nine pediatric patients with Crohn’s disease, one patient received dual therapy with infliximab and anakinra. However, the outcome in this patient was difficult to assess based on reported results (Goyal 2020 S122) [129].

There is also preliminary data to suggest that IL-1 antagonists may be useful in defects of the IL-10 pathway. IL-10 is an immunoregulatory cytokine, which mediates several anti-inflammatory pathways via its receptor. Mutations in the receptor encoding genes *IL10RA* and *IL10RB* can lead to the development of severe, refractory infantile VEO-IBD [34,130]. Anakinra has been used as a steroid-sparing agent to bridge a small number of patients with *IL10RA* mutations to stem cell transplant, based on work showing that IL-10 deficient macrophages produce higher levels of IL-1b and lead to downstream inflammasome activation [131].

#### Pharmacologic Considerations and Therapeutic Drug Monitoring for IL-1 Inhibitors

Anakinra and canakinumab both require baseline screening for TB and hepatitis B in addition to baseline and periodic hematologic, renal, and hepatic monitoring. There is no drug level monitoring required (Table 3).

### 5.6. Abatacept

Abatacept is a cytotoxic T lymphocyte antigen-4 (CTLA4) immunoglobulin fusion drug. CTLA4 is an inhibitory immune checkpoint protein that is expressed on FOXP3+ T-regulatory cells. Abatacept is used as a targeted therapy for LRBA deficiency, which is marked by very low CTLA4 expression and presents with enteropathy and other systemic manifestations [3,11,12,132]. In a study of 22 children with LRBA deficiency who were treated with abatacept, 11 achieved complete remission, and three had partial remission. The majority were also able to stop steroids and other immunosuppressive agents [133]. Abatacept was also used with some success in the case of a child who developed post-heart transplant autoimmune enteropathy consistent with Omenn syndrome, marked by the presence of autoreactive oligoclonal T cells [134]. Overall, the use of abatacept in VEO-IBD has been quite limited and overall remission rates are not reported.

#### Pharmacologic Considerations and Therapeutic Drug Monitoring for Abatacept

Patients who are initiated on abatacept therapy require baseline TB and hepatitis B screening, in addition to assessment for increased risk of infection and possible hypersensitivity reactions. No drug level monitoring is required (Table 3) [101].

### 5.7. IL-18 Antagonists

IL-18 is another downstream product of the proinflammatory cascade produced by inflammasome activation. IL-18 production is a hallmark of several monogenic immunodeficiencies that produce IBD symptoms, including macrophage activation syndrome (MAS) secondary to *NLRC4* mutation, as well as mutations in X-linked inhibitor of apoptosis (XIAP). *NLRC4* encodes a key component of the inflammasome complex. Patients with this mutation develop macrophage activation syndrome and severe neonatal-onset enterocolitis [135,136]. This disease is marked by very high levels of IL-18. There are not currently any IL-18 antagonists approved for use in humans; however, there are ongoing clinical trials of several recombinant IL-18 binding proteins (rhIL-18BP). These have occasionally been made available by compassionate use protocols for patients with IL-18-related disorders [137]. There is a pediatric phase II trial of MAS825, an anti-IL-1b/IL-18 monoclonal antibody, for patients with *NLRC4* gain-of-function mutations (US NLoM NCT04641442). XIAP also involves inflammasome activation and significant elevation in IL-18 [10]. There is an ongoing phase III trial at Cincinnati Children’s Hospital studying the rhIL-18BP drug tadekinig alfa in patients with both XIAP and NCLR4 mutations (US NloM NCT03113760). As these medications are still experimental, there is no information available about side effects, screening, or drug level monitoring.

## 6. Empiric Therapies

Therapy for inflammatory bowel diseases historically involved targeting immune dysregulation through systemic immunosuppression using glucocorticoids, non-specific immunomodulators, such as azathioprine and methotrexate, and calcineurin inhibitors. In recent years, the biologic therapies and small molecules that target specific immune pathways previously discussed have reduced reliance on these medications.

### 6.1. Immunomodulators

The immunomodulators azathioprine (AZA) and methotrexate can be used as monotherapy or in combination with biologics to treat IBD. AZA blocks de novo purine synthesis and thus arrests the development of immune cells. Methotrexate is a folate antagonist, which exerts its immunomodulatory action via several pathways: inhibition of purine and pyrimidine synthesis, production of reactive oxygen species, and modulation of cytokine production [138]. In one case report, an infant who presented with neonatal liver failure and refractory IBD-like pancolitis due to a pathogenic cytosolic isoleucyl-tRNA synthetase mutation showed improvement after initiation of subcutaneous methotrexate. CD4+ memory T-cell secretion of proinflammatory cytokines, including IL-2, IL-5, IL-9, and IL-13 were elevated in this patient [139]. Methotrexate has been shown to be an inhibitor of cytokine production in activated T cells in in vitro studies of patients with rheumatoid arthritis, which suggests a possible mechanism for the noted improvement [140]. One meta-analysis showed that, for patients with Crohn’s disease, 37% of the studied pediatric population was able to achieve 12 month remission with methotrexate monotherapy [56]. In a retrospective study conducted in Canada, investigators evaluated the use of methotrexate monotherapy in pediatric patients with Crohn’s disease, ulcerative colitis, and indeterminate colitis. Clinical remission was achieved in 16% of the Crohn’s disease group compared to 7% in patients with UC or IBD-U. Overall, long-term remission rates with methotrexate in this population was low [141]. When evaluating data supporting the use of azathioprine, the use of genetic testing becomes important due to the role of thiopurine methyltransferase, which is required for conversion of AZA into its active metabolites. Reduced activity of this enzyme can result in increased risk of myelosuppression and hepatotoxicity. Out of 41 pediatric patients evaluated in one retrospective study conducted in England, 12 patients required dose increases to achieve clinical remission. A total of 28 patients did not require AZA dose adjustments and achieved [142].

#### 6.1.1. Pharmacologic Considerations and Therapeutic Drug Monitoring for Immunomodulators

Young children with VEO-IBD may require higher doses of AZA to achieve therapeutic levels, which leads to increased risk of hepatoxicity and bone marrow failure [10,143]. As previously mentioned, azathioprine therapy has also been linked with hepatosplenic T-cell lymphoma, and its use for longer than two years is not recommended [10].

#### 6.1.2. Time to Therapeutic Effect

When evaluating time to clinical response, adult data have shown that in Crohn’s disease it may take between two weeks and nine months to achieve clinical remission with azathioprine [79,106]. In pediatric IBD, maximum effectiveness in Crohn’s disease has been reported to take between eight and sixteen weeks [49] (Table 4).

### 6.2. Calcineurin Inhibitors

Calcineurin inhibitors (cyclosporine A and tacrolimus) work by suppressing transcription of IL-2, TNFα, and interferon-c in T cells, and have been used for induction of remission in steroid-refractory pediatric IBD [144,145]. While pediatric studies have shown response rates of 60–80% in older children, their use in the VEO-IBD population has had mixed results [146]. One review of XIAP patients treated with tacrolimus found that 92% of patients were refractory to treatment with combination corticosteroids and tacrolimus, as well as cyclosporine and AZA [147].

#### 6.2.1. Pharmacologic Considerations and Therapeutic Drug Monitoring for Calcineurin Inhibitors

Tacrolimus and cyclosporine have been used to treat severe colitis in children and guidelines suggest their use as an alternative second line agent after failure to respond to steroids [71]. These medications are typically used to bridge to agents with a longer time to effect, such as vedolizumab, ustekinumab, or thiopurines. Tacrolimus has been dosed as 0.1 mg/kg orally every 12 h as part of induction therapy, targeting a goal trough level of 10–15 ng/mL [71]. One study reported decreasing the goal trough goal to 5–7 ng/dL once therapeutic remission was achieved [71]. Cyclosporine can be given as a continuous infusion, with one study comparing 4 mg/kg/day with goal trough range of 250–350 ng/mL to 2 mg/kg/day with goal levels between 150–250 in adult patients. Authors found that higher dosing was not shown to have any additional clinical benefit [73,74]. In the pediatric literature, the goal trough level for induction when using 2 mg/kg/day via continuous infusion is recommended to be between 150–300 ng/mL, and, once remission is achieved, this can be decreased to 100–200 ng/mL [71]. For oral dosing, one retrospective study with 14 children who received calcineurin inhibitors therapy (six of which were given cyclosporine) evaluated 4–8 mg/kg/day of cyclosporine and found that a goal trough range of 150–300 ng/mL was effective [75]. A second study with 28 children evaluated 5 mg/kg/day oral dosing while targeting trough levels between 150–250 ng/mL [76](Table 2). There are several different preparations of oral cyclosporine on the market, including Sandimmune and Neoral. Caution should be used when selecting an agent as these are not interchangeable products; Neoral is a microemulsion with better bioavailability [148]. Both tacrolimus and cyclosporine possess the risk of causing nephrotoxicity and serum electrolyte derangements, for which close monitoring of renal function in addition to potassium, magnesium, and phosphate are recommended (Table 3).

#### 6.2.2. Time to Therapeutic Effect

The reported time to see clinical effect from calcineurin inhibitors is relatively quicker than other classes of medications used to treat steroid refractory ulcerative colitis in the pediatric population. With enteral tacrolimus response can be seen in just 14 days [72]. Oral cyclosporine showed clinical response within seven to fifteen days when using the enteral dosing scheme of 5 mg/kg/day [76,111] (Table 4).

## 7. Non-Pharmacologic Therapies

Several non-pharmacologic therapies are employed in treating monogenic and VEO-IBD, including nutrition, surgery, and hematopoietic stem cell transplantation (HSCT). HSCT is the only non-pharmacologic therapy that can be targeted at specific monogenic defects; however, it is important to note that it may worsen intestinal disease in some monogenic disorders, and thus accurate genetic diagnosis is critical prior to initiating therapy or considering HSCT. HSCT is curative in SCID, CGD, DOCK8 deficiency, IPEX syndrome, Wiskott-Aldrich syndrome, LRBA deficiency, and LAD1, and it improves intestinal disease in STAT-1 and STAT-3 GOF syndromes, IL-10 deficiency, and IL-10R deficiency [3,41]. However, it should be used with caution in patients with epithelial barrier defects, such as X-linked recessive ectodermal dysplasia with immunodeficiency. While HSCT cures the defect in immune cells, it does not affect the defective intestinal epithelial cells, and thus it may not improve intestinal disease. In some cases, HSCT can worsen or lead to de novo IBD symptoms in these patients after transplant [149]. The risks and benefits of HSCT should be carefully weighed in all patients with monogenic IBD, as HSCT can have severe consequences, including sepsis, graft versus host disease, secondary malignancy, and death [3].

Nutritional therapy with exclusive enteral nutrition (EEN) is used as a steroid-sparing strategy for induction of remission in pediatric Crohn’s disease and has been shown to be as effective as corticosteroids [150,151]. The mechanism of its action remains unclear, though is thought to involve modulation of the gut microbiome. It can be used safely in VEO-IBD, and in one small study, its use led to clinical remission in two infants [152]. Surgery can improve symptoms and quality of life in treatment-refractory monogenic and VEO-IBD, but it does not target underlying genetic defects. There is some evidence to suggest that VEO and monogenic IBD patients are more likely than older patients to require surgery due to the refractory nature of their disease [10,153], and thus referral to a center with experienced pediatric surgeons is needed.

## 8. Conclusions

The recent development of new medications that target specific immune pathways has revolutionized the treatment of inflammatory immune diseases, and it is poised to include monogenic IBD. Targeted therapy may allow for decreased reliance on systemic immunosuppressants, thereby reducing the likelihood of developing side effects or drug toxicity. When considering pharmacologic options for managing the VEO-IBD and monogenic IBD populations specifically, there is currently limited published data to support the use of these medications. Current practice relies on observational case studies, retrospective reviews, and ongoing clinical trials, or extrapolation from the polygenic IBD population. However, navigating the unknown in pediatric medicine is not foreign to those caring for these patients.

Large-scale randomized control trials may never be feasible in this population due to the rarity of these disorders. However, the field of oncology has lately been revolutionized by the advent of functional precision medicine. This allows for a single individual’s tumor cells to be directly inoculated with drugs and allows for instant, personalized profiling of response to therapy [154]. While such strategies do not yet exist for IBD, there are currently some promising research protocols aimed at predicting response to therapies, including cytokines and gene, microRNA, and microbial signatures [155]. When evaluating the role of precision medicine in the diagnosis of these patients, a high index of suspicion leading to early genetic diagnosis is of paramount importance in monogenic IBD, as patients with these disorders require complex care delivered by multidisciplinary teams in order to have the best possible outcomes. Early recognition and diagnosis allow children maximal opportunity for normal growth and development with minimal impairment due to treatment effects, and in some cases, a cure via stem cell transplantation. As more disease-causing genes are identified and new medications are developed, children with monogenic IBD will benefit from their position at the forefront of precision medicine.

## Figures and Tables

**Table 1:** Summary of monogenic etiologies of VEO-IBD.

Type of Disorder	Representative Syndromes	Genes
Epithelial cell defects	TTC7A deficiency NEMO deficiency Dystrophic epidermolysis bullosa ADAM-17 deficiency	*TTC7A* *IKBKG* *COL7A1* *ADAM17*
Phagocytic defects	Chronic granulomatous diseaseLeukocyte adhesion deficiency	*CYBA, CYBB, NCF1, NCF2, NCF4* *ITGB2*
Defects of adaptive immunity (T and B cells)	Wiskott-Aldrich syndrome Bruton agammaglobulinemia Loeys-Dietz syndrome Severe Combined Immunodeficiency (SCID)	*WAS* *BTK* *TGFBR1, TGFBR2* *ZAP70, RAG2, 1L-2RG, LIG4, ADA*
T regulatory defects	IPEX IPEX-like syndrome CTLA4 deficiency LRBA deficiency	*FOXP3* *STAT1, STAT3, JAK1, IL-2RA* *CTLA4* *LRBA*
IL-10 pathway defects	IL-10 IL-10R	*IL10* *IL10RA, IL10RB*
Hyperinflammatory or autoinflammatory defects	X-linked lymphoproliferative syndrome Hermansky-Pudlak syndrome Mevalonate kinase deficiency	*XIAP* *HPS1, HPS4, HPS6* *MVK*

**Table 2:** Therapeutic Drug Monitoring (TDM) for Monoclonal Antibodies and Calcineurin Inhibitors in IBD.

Drug	Timing of Trough Levels	Goal Trough Level (mcg/mL)
Infliximab	Induction Phase (with 2nd and 3rd infusions) Maintenance Phase (prior to 4th infusion)	Pediatric General (Maintenance): >5 mcg/mL [49] Pediatric Perianal Fistulizing Disease (Maintenance): >12.7 mcg/mL [58] Pediatric Luminal Crohn’s Disease (Induction): Infusion two and Infusion three: trough level of ≥29 mcg/mL at the 2nd infusion and ≥18 mcg/mL at 3rd infusion were strongly associated with improve outcomes early on in therapy and higher levels during maintenance phase [59] Pediatric Luminal Crohn’s Disease (Induction/Maintenance): ≥25 mcg/mL at infusion two (week 2) and ≥15 mcg/mL at infusion three (week six) were associated with better outcomes [49] Pediatric Ulcerative Colitis (Induction): trough level of ≥33 mcg/mL prior to the second dose was associated with clinical remission at eight weeks of therapy [60]. PK analysis showed that a trough level of ≥41.1 mcg/mL at week eight was associated with higher clinical remission, mucosal healing, and clinical response based on Mayo scoring [61,62]
Adalimumab	Maintenance Phase (prior to 3rd injection)	Pediatric General (Maintenance): >7.5 mcg/mL for endoscopic healing at week 8 [49] Pediatric Crohn’s Disease (Maintenance): levels of >22.5 mcg/mL at week four and trough levels > 12.5 mcg at week eight were associated with prediction of clinical remission at week 24 [63]
Vedolizumab [64]	Lack of data to suggest optimal timing	Lack of data to suggest optimal goal trough levels [49] Vedolizumab trough concentrations are associated with clinical response and dose escalation may be required to maintain this response [65] Pediatric IBD: average trough levels of 32.1 mcg/mL at week 2 of therapy and 29.9 mcg/mL at week six were observed in a cohort of 22 pediatric patients.; when delineated by type of IBD, trough levels in patients with UC/IBD-U were higher than in those with CD [66] Dose escalation in adult IBD: vedolizumab trough level of <7.4 mcg/mL at eight weeks of therapy indicated probable response to dose escalation (dosing frequency of up to every four weeks) [65] Adult study (maintenance): trough level of 33–37 mcg/mL at week 6,15–20 mcg/mL after induction (week 14) and 10–15 mcg during maintenance phase was associated with improved clinical outcomes [67]
Ustekinumab	Lack of data to suggest optimal timing	Single case report in which a VEO-IBD patient achieved clinical remission with a trough level of 6 mcg/mL after 18 months of treatment (therapy started at age seven yrs) [68] Adult studies: In one CD study: trough level of at least 2 mcg/mL at week eight was associated with clinical response to induction by week 16 of therapy [69] Another study indicated a trough of at least 4.5 mcg/mL by week 26 of therapy to be associated with better endoscopic response in CD Trough level of 0.8–1.4 mcg/mL was associated with clinical remission during the maintenance phase of the STARDUST trial (assessing CD patients) [67,70] Trough level of 3–7 mcg/mL at week eight and 1–3 mcg/mL during maintenance for UC and CD [67]
Tacrolimus Cyclosporine		Tacrolimus (pediatric): for severe colitis in children: 0.1 mg/kg/dose q12 with goal trough of 10–15 ng/ ml for induction therapy; one study reported decreasing the goal trough to 8–10 ng/dL once frank blood was absent; initial response rate has been shown to be similar to that of cyclosporine treated patients. Some guidelines recommend decreasing to 5–7 ng/mL once remission achieved [71,72] CSA (adult): for UC in an RCT comparing 4 mg/kg/day via continuous infusion (goal trough range of 250–350 ng/mL) to 2 mg/kg/day of IV therapy (goal 150–250 ng/mL): high dose CSA was not shown to have additional clinical benefit compared to lower dosing [73,74] CSA IV (pediatric): goal trough during induction with 2 mg/kg/day continuous infusion: 150–300 ng/mL and once remission achieved, may decrease to 100–200 ng/mL [71] CSA PO (pediatric): goal trough of 150–300 ng/mL for 4–8 mg/kg/day of oral dosing in a retrospective study with 14 children, of which six were treated with CSA [75]; Second study with 28 patients started on 5 mg/kg/day of oral CSA while targeting goal trough levels of 150–250 ng/mL [76]

Abbreviations: CSA: cyclosporine; PK: pharmacokinetics; IBD: inflammatory bowel disease; CD: Crohn’s Disease; UC: Ulcerative Colitis: PO: by mouth; IV: intravenous.

**Table 3:** Baseline Screening and Monitoring for Immune-Targeted Therapies.

Medication	Mechanism of Action	Screening/Baseline Labs	Black Box Warnings/Common ADRs/Monitoring
Azathioprine 6-MP [89,90,91,92,93]	Purine analog, blocks DNA replication and proliferation of T-cells; possible inhibition of CD28 T-cell co-stimulation	TPMT, NUDT15 prior to starting treatment LFTs, CBC + diff: check at baseline, then every one to two weeks during the first month, then every three months	BBW: Malignancy (hepatosplenic T cell lymphoma) Hematologic (leukopenia, thrombocytopenia, anemia) Pancreatitis (would warrant discontinuation of the drug) Gastrointestinal symptoms (nausea/vomiting), hepatotoxicity
Infliximab Adalimumab [49,94,95]	Anti- TNFα	TB status, hepatitis B, varicella, vaccination status; creatinine, fecal calprotectin, CRP, LFTs prior to starting treatment	BBW: Malignancy, TB, infection Infliximab: infusion related reactions (cutaneous, psoriatic rash, elevated transaminases, infection Adalimumab: injection site reaction, infection
Vedolizumab [49,64,88]	Anti-α4β7 integrin; blockade of MAdCAM-1 directed lymphocyte traffic to intestinal Peyer’s patches	TB status prior to therapy initiation, LFTs prior to starting treatment	BBW: N/A Low percentage of ADRs reported which have led to therapy discontinuation (5–10%) ADA production is uncommon Not associated with increased risk of malignancy or opportunistic infections Hypersensitivity reactions may occur during infusion
Ustekinumab [96]	Anti-IL12 and IL-23	TB, hepatitis B, hepatitis C, HIV screening prior to starting therapy, CBC with differential, CMP, reversible posterior leuko-encephalopathy syndrome, ADAs	BBW: N/A Hypersensitivity reactions may occur during infusion Increased risk of infection
Tacrolimus Cyclosporine [71,72,76,97,98]	Suppression of IL-2, TNFα, and interferon-c production in T cells	Serum electrolytes (K, Mag, Phos), LFTs, renal function, blood pressure, glucose: should be checked three timesper week upon therapy initiation. Serum cholesterol recommended before starting treatment with CSA. Frequency of lab checks may be spaced once stability has been shown	BBW for Tacrolimus: increased risk of infection and malignancy BBW for CSA: increased infection, development of neoplasia Tacrolimus: infection, malignancy (lymphoma, skin related cancers) CSA: increased risk of infection, nephrotoxicity, and hypertension. Dosage forms may affect drug concentrations and bioavailability Tacrolimus and CSA: Nephrotoxicity, serum electrolyte derangements (K, Mag, Phos), immune suppression, azotemia, gingival hyperplasia, hirsutism, tremor DDIs: tacrolimus and cyclosporine metabolized by CYP3A enzymes (thorough drug interaction checking recommended when initiating or discontinuing medications for patients on calcineurin inhibitors)
Anakinra [99] Canakinumab [100]	IL-1 antagonist	TB and hepatitis B status prior to starting therapy, CBC with differential, LFTs	BBW: N/A Increased risk of infection Signs of hypersensitivity reactions CBC with differential q3 months up to one year, renal function
Abatacept [101]	Cytotoxic T lymphocyte antigen-4 (CTLA4) immunoglobulin fusion molecule	TB and hepatitis status prior to starting therapy	BBW: N/A Hypersensitivity reactions Increased risk of infection
Tofactinib [102] Ruxolitinib [103]	JAK 1/2 and 1/3 inhibitor	Tofacitinib: - TB status prior to starting therapy - CBC with differential (hemoglobin, neutrophil count, lymphocyte count, platelets) - Heart rate and blood pressure - Renal function and LFTs Ruxolitinib: CBC, renal function, hepatic function	BBW: malignancy (including solid tumors and lymphoma), higher infection risk, increased thrombotic risk Tofacitinib: lipid abnormalities (dose- dependent), renal and hepatic impairment. - Lymphocyte count q3 months - CBC with differential (hemoglobin, neutrophil count, platelet count) every four to eight weeks, then every three months - Lipids: four to eight weeks after starting treatment Ruxolitinib: CBC every two to four weeks until on stable dose, lipid count eight to twelve weeks after starting therapy, hepatic and renal function every two to four weeks until on stable dose (extrapolated from treatment of GVHD) DDIs: Tofacitinib and Ruxolitinib are major CYP3A4 substrates (thorough drug interaction checking recommended when initiating or discontinuing medications for patients on JAK inhibitors)

TPMT: thiopurine methyltransferase; BBW: black box warning; ADR: N/A: not applicable; adverse drug reaction. TB: tuberculosis; ADA: anti-drug antibody; CRP: C-reactive protein; HIV: human immunodeficiency virus; CBC: complete blood count; CMP: complete metabolic panel; K: potassium; mag: magnesium; phos: phosphate; LFTs: liver function testes; DDI: drug–drug interaction; IV: intravenous; PO: by mouth; RCT: randomized controlled trial; CSA: cyclosporine; SrCr: serum creatinine.

**Table 4:** Time to Therapeutic Effect for Immune-Targeted Therapies.

Medication	Time to Full Therapeutic Effect/Clinical Remission
Azathioprine 6-MP	CD (adult): May take a minimum of eight weeks to achieve clinical remission; wide range reported ranging from two weeks to nine months [79,106] UC (adult): typical range of three to six months to see clinical and endoscopic response [79,107] UC and CD (pediatric): pediatric IBD network showed remission within 180 days of starting thiopurine therapy, with approximately 50% of patients reporting sustained steroid free remission at six months [108]. Range of eight to sixteen weeks has been reported to reach maximum effectiveness in CD [49]
Infliximab Adalimumab	Infliximab: response is variable and patient dependent; requires monitoring of trough levels and ADAs. Many patients may require dose escalation within the first year of treatment [77,78,79] CD (adult): clinical response and remission for infliximab reported in one study to take eight to nine days, with up to 81% of patients with clinical response rates after four weeks of therapy [77,78,79,109] UC (adult): could be dose dependent; for acute severe colitis could expect response to infliximab to be within seven days [79,110] CD (pediatric): 1/3 of patients are reported to lose response within one year (secondary loss of response); immunomodulator use has made a clinical difference in this loss of clinical response [50] UC (pediatric): time to clinical response varies; infliximab was found to be safe and effective with a response by week eight of therapy in nearly 75% of pediatric patients with moderate to severe UC [82] Adalimumab: Significant patient variability: clinical remission rates have ranged from two to twenty-four weeks in the literature in adults CD (adult): initial response and remission for adalimumab reported within a few weeks; endoscopic remission would be longer, with response time ranging from 12–52 weeks [79,80] UC (adult): clinical remission with adalimumab was seen as early as four weeks [79,81] CD and UC (pediatric): wide range of clinical response times reported in the literature; clinical response seen ranging from two weeks to two years [83]
Vedolizumab	Vedolizumab may be more effective in UC vs. CD. Lower percent of patients with mucosal healing shown in patients with CD compared to UC [49] Response may take up to 16 weeks (some studies reported therapeutic onset to be between eight and twelve weeks); may require a bridging agent such a steroids until clinical response is seen [49] 76% of UC patients and 42% of CD in a retrospective study who had failed anti-TNF therapy achieved clinical remission at week 14 [105] Overall lower immunogenicity when compared to adalimumab or infliximab and lower percentage of patients with ADAs compared to anti-TNFs [104] Patients without prior biologic exposure, less severe disease, and early response to vedolizumab may have higher rates of endoscopic and clinical remission [104]
Ustekinumab	Some evidence to show better response and higher rate of clinical remission in patients who are biologic non-naïve In adult trials, clinical response was seen after eight weeks of treatment. Some studies suggest a timeframe of up to 14 weeks for clinical response, and up to six months for endoscopic remission Addition of immunomodulator therapy does not appear to affect immunogenicity
Tacrolimus Cyclosporine	Tacrolimus: Generally used in steroid refractory UC Response may be seen within the first 14 days after tacrolimus initiation (enteral dosage form). May be used as a bridge while waiting for biologic therapy such as vedolizumab to take effect (this is post steroid and anti-TNF therapy failure); may take between eight and twelve weeks to see effects of vedolizumab ECCO Guidelines suggested role in adult UC and proctitis: tacrolimus in patients with refractory UC extending beyond the rectum. Topical tacrolimus: clinical response may be seen after four weeks of therapy. Limited evidence for use of tacrolimus enemas in pouchitis CSA IV (adult): would have a predicted response in at least 50% of patients after four to five days of treatment, with full response seen by 14 days. Would typically begin with continuous infusion and then transition to enteral dosing [73] CSA PO (pediatric): in a study of 28 children, clinical response was seen within seven to fifteen days after starting an enteral dose of 5 mg/kg/day with a goal target of 150–250 ng/ml [76,111]

Abbreviations: CSA: cyclosporine; IBD: inflammatory bowel disease; CD: Crohn’s disease; UC: ulcerative colitis; ADA: anti-drug antibodies; PO: by mouth; IV: intravenous.
